# Analysis of 12/15-lipoxygenase metabolism of EPA and DHA with special attention to authentication of docosatrienes

**DOI:** 10.1016/j.jlr.2021.100088

**Published:** 2021-05-20

**Authors:** Jing Jin, William E. Boeglin, Alan R. Brash

**Affiliations:** Department of Pharmacology and the Vanderbilt Institute of Chemical Biology, Vanderbilt University, Nashville, TN, USA

**Keywords:** docosatrienes, eicosanoids, enzymology, leukotrienes, lipid biochemistry, lipoxygenase, LC, omega-3 fatty acids, specialized proresolving mediators, spectrometry, 14*S*-HPDHA, 14*S*-hydroperoxide-DHA, 15*S*-HPEPE, 15*S*-hydroperoxide of EPA, 16,17-DTA_6_, 16,17-docosatriene A_6_ (analogue of LTA_4_), 16*S*,17*S*-*trans*-epoxy-4*Z*,7*Z*,10*Z*,12*E*,14*E*,19*Z*-docosahexaenoic acid, 17*S*-HPDHA, 17*S*-hydroperoxide-DHA, LOX, lipoxygenase, LTA, leukotriene A, LTA_4_, leukotriene A_4_, LTB_4_, leukotriene B_4_, MaR1, maresin R1, MeOH, methanol, NPD1, neuroprotectin D1, PD1, protectin D1, RP-HPLC, reversed-phase HPLC, SP-HPLC, straight-phase HPLC

## Abstract

A proposed beneficial impact of highly unsaturated “fish oil” fatty acids is their conversion by lipoxygenase (LOX) enzymes to specialized proresolving lipid mediators, including 12/15-LOX products from EPA and DHA. The transformations of DHA include formation of docosatrienes, named for the distinctive conjugated triene of the double bonds. To further the understanding of biosynthetic pathways and mechanisms, herein we meld together biosynthesis and NMR characterization of the unstable leukotriene A (LTA)-related epoxide intermediates formed by recombinant human 15-LOX-1, along with identification of the stable enzymatic products, and extend the findings into the 12/15-LOX metabolism in resident murine peritoneal macrophages. Oxygenation of EPA by 15-LOX-1 converts the initial 15*S*-hydroperoxide to 14*S*,15*S*-*trans*-epoxy-5*Z*,8*Z*,10*E*,12*E*,17*Z*-EPA (appearing as its 8,15-diol hydrolysis products) and mixtures of dihydroperoxy fatty acids, while mainly the epoxide hydrolysis products are evident in the murine cells. DHA also undergoes transformations to epoxides and dihydroperoxides by 15-LOX-1, resulting in a mixture of 10,17-dihydro(pero)xy derivatives (docosatrienes) and minor 7*S*,17*S*- and 14,17*S*-dihydroperoxides. The 10,17*S*-dihydroxy hydrolysis products of the LTA-related epoxide intermediate dominate the product profile in mouse macrophages, whereas (neuro)protectin D1, the leukotriene B_4_-related derivative with *trans*,*trans*,*cis* conjugated triene, was undetectable. In this study, we emphasize the utility of UV spectral characteristics for product identification, being diagnostic of the different double bond configurations and hydroxy fatty acid functionality versus hydroperoxide. LC-MS is not definitive for configurational isomers. Secure identification is based on chromatographic retention times, comparison with authentic standards, and the highly distinctive UV spectra.

The long-standing interest in the therapeutic benefits of dietary fish oil fatty acids—EPA and DHA—is extended in more recent times to the potential anti-inflammatory/proresolving effects of their lipoxygenase (LOX)-derived metabolites (1, 2). Aside from direct effects of the omega-3 fatty acids themselves, the daily intake of high doses might act partly by diverting in vivo fatty acid metabolism away from the omega-6 arachidonic acid, thus decreasing production of proinflammatory prostaglandins and leukotrienes, the “bad guys” in controlling inflammation (3, 4). On the other hand, many studies appearing in the past two decades support the important roles played directly by potent local bioactive mediators derived from omega-3 PUFA. The series of molecules tagged as resolvins and protectins and “specialized proresolution mediators” has sparked great interest on account of their potent biological activities documented in the fields of inflammation, neuroscience, diabetes, and cancer (e.g., Refs. (1, 2, 5, 6, 7, 8)).

Modeled on the 5-LOX pathway of leukotriene biosynthesis and central to the production of the spectrum of the EPA- and DHA-derived metabolites is the production of an unstable leukotriene A (LTA)-related epoxide as a biosynthetic intermediate (e.g., Ref. (9)). On account of the extreme instability of LTA-related epoxides in neutral aqueous media, their biosynthesis is established primarily by analysis of their hydrolysis products; in aqueous media, the production of dihydroxy derivatives, and in “trapping” studies using an excess of alcohol to terminate reaction, the production of hydroxymethoxy derivatives (10). Notwithstanding the technical challenges, leukotriene A4 (LTA_4_) itself has been isolated from leukocyte incubations (11), and later, we prepared LTA_4_ and its 5,6-*cis*-epoxide analogue using recombinant 5-LOX enzyme (12). Herein, we applied the methodology using recombinant human 15-LOX-1 to preparation of the LTA-related epoxides from *n*-6 hydroperoxides of EPA and DHA, the latter and 22:5ω3 analogue also prepared by total chemical synthesis (13, 14, 15, 16).

Also modeled on the 5-LOX pathway is the reported hydrolysis of LTA-related epoxides in 15-LOX/DHA metabolism to a dihydroxy derivative exhibiting a *cis,trans,trans* conjugated triene chromophore (9). This is a special type of transformation that does not occur through nonenzymatic hydrolysis of LTA-related epoxides. In the leukotriene pathway, the epoxide LTA_4_ is enzymatically hydrolyzed to leukotriene B_4_ (LTB_4_), the prototypical lipid mediator with the unusual 5*S*,12*R*-dihydroxy configuration along with a *cis,trans*,*trans* conjugated triene (Scheme 1A). Reported analogous examples in 12/15-LOX metabolism of DHA are the conversions of LTA-related 16,17-*trans*-epoxy-DHA (designated herein as docosatriene A_6_) to protectin D1 (PD1), a 10*R*,17-diol with 11*trans*,13*trans*,15*cis* triene (Scheme 1B) (17), and more recently, the production of maresin R1 (MaR1), formed from the analogous 13,14-epoxy intermediate as a 7*R*,14*S*-diol also with a *trans*,*trans*,*cis* triene chromophore (18, 19).

**Scheme 1:**
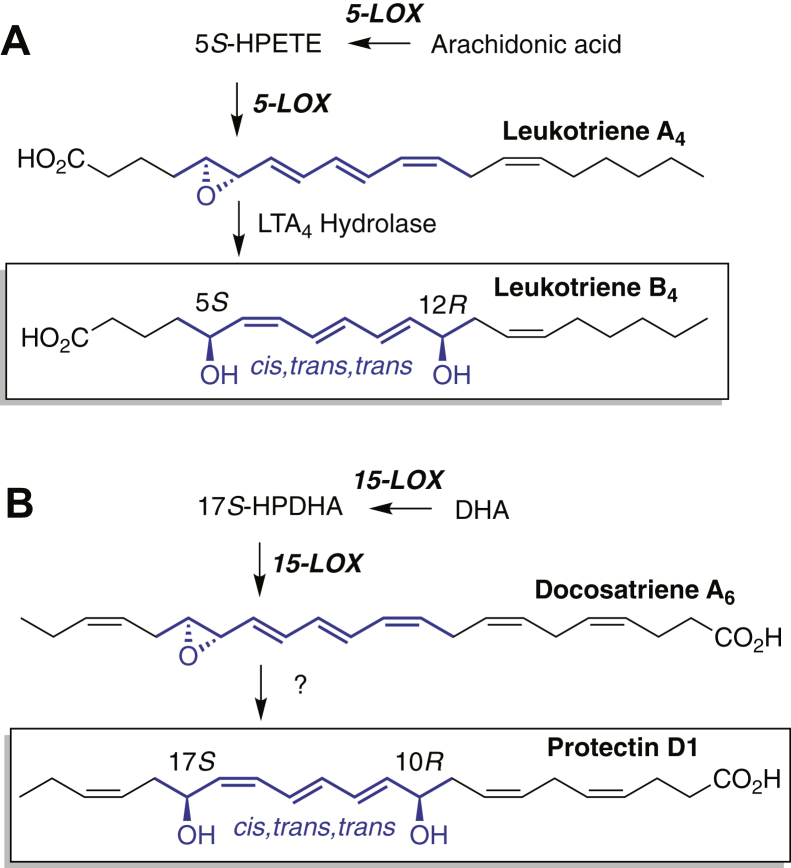
Parallels in (A) LTB_4_ and (B) protectin D1 biosynthesis. LTB_4_, leukotriene B_4_.

From arachidonic acid, multiple 5,12-dihydroxy isomers of LTB_4_ can be formed, and by which criteria do we identify these biosynthetic products? We rely on distinctive HPLC retention times associated with the mass ions for 5,12-diols and on the characteristic UV spectra. Indeed, 40 years ago, the distinctive UV spectra of different isomers were established as central to the analysis (20, 21). With the EPA- and DHA-derived LOX products, quality UV spectra are reproducible and distinctive, and this criterion is among those used to identify products in the studies presented here. Herein, we meld together biosynthesis and NMR characterization of the LTA-related unstable epoxide intermediates, with analysis of their biosynthesis along with other enzymatic products by recombinant 15-LOX-1, and extend the findings into the metabolism in inflammatory cells. Most of these studies were completed as a PhD thesis (22) and augmented more recently with the availability of synthetic PD1 and MaR1 as authentic standards.

## Materials and methods

### Materials

DHA and EPA were purchased from NuChek Prep, Inc (Elysian, MN). Soybean LOX-1 (lipoxidase, type V) was purchased from Sigma. C57BL/6NCrl mice (6–8 weeks) were purchased from Charles River Laboratories. Zymosan A was purchased from Sigma. We thank Professor Trond Hansen for providing a sample of synthetic PD1. PD1 and MaR1 were also purchased from Cayman Chemical.

### Synthesis and purification of fatty acid hydroperoxides

The 15*S*-hydroperoxide of EPA (15*S*-HPEPE) and 17*S*-hydroperoxide-DHA (17*S*-HPDHA) were prepared by the reaction of the fatty acids with the commercially available soybean LOX-1. 14*S*-hydroperoxide-DHA (14*S*-HPDHA) was prepared by the reaction of DHA with mouse platelet-type 12*S*-LOX. The purification of fatty acid hydroperoxides was achieved by straight-phase HPLC (SP-HPLC) using a solvent of hexane/isopropyl alcohol/glacial acetic acid, 100/1/0.02, by volume, and they were quantified by UV spectroscopy using an extinction coefficient of 25,000 M^−1^ cm^−1^ for the conjugated diene chromophore.

### Bacterial expression and purification of human 15-LOX-1

The cDNA of human 15-LOX-1 was subcloned into the pET3a vector (with an N-terminal His6 tag), and the protein was expressed in BL21 cells. A typical preparation of a 100 ml culture was carried out as follows: 100 ml of 2xYT medium containing 100 μg/ml ampicillin was inoculated with a single colony of human 15-LOX-1-His in BL21 cells and grown at 37°C at 250 rpm till an absorbance at 600 nm reached 0.8. IPTG (0.5 mM) was then added to the culture, which was grown at 16°C, 220 rpm for 4 days. On day 4, the cells were spun down at 5,000 *g* for 20 min in a Beckman Avanti J-25I centrifuge, washed with 40 ml of 50 mM Tris, pH 7.9, pelleted again at 5,000 *g* for 20 min, and resuspended in 10 ml of 50 mM Tris, pH 8.0, 500 mM NaCl, 20% glycerol, and 100 μM PMSF. The spheroplasts were then sonicated five times for 10 s using a model 50 Sonic Dismembrator (Fisher Scientific) at a setting of 5. CHAPS detergent was added at a final concentration of 1% (w/w), and the sample was kept on ice for 20 min. The resulting membranes were spun down at 5,000 *g* for 20 min at 4°C. The human 15-LOX-1 activity was present in the supernatant. The supernatant was loaded on a nickel-nitrilotriacetic acid column (0.5 ml bed volume; Qiagen) equilibrated with 50 mM Tris buffer, pH 8.0, and 500 mM NaCl. The column was then washed with the equilibration buffer, and the nonspecific bound proteins were eluted with 50 mM Tris buffer, pH 8.0, 500 mM NaCl, and 50 mM imidazole. The human 15-LOX-1 was then eluted with 50 mM Tris buffer, pH 8.0, 500 mM NaCl, and 250 mM imidazole. Fractions of 0.5 ml were collected and assayed for the LOX activity. The positive fractions were dialyzed against 50 mM Tris buffer, pH 7.5, and 150 mM NaCl. The purity of the enzyme preparations was determined by SDS-PAGE and Coomassie blue staining; the prominent band of h15-LOX-1 accounted for about 80% of the total protein.

### Biphasic reaction conditions for preparation of LTA-related epoxides

Enzyme reactions were performed at 0°C, with the fatty acid hydroperoxide substrate initially in hexane (5 ml, bubbled for 30 min prior to use with argon to decrease the O_2_ concentration, and containing ~200 μM substrate) layered over recombinant 15-LOX-1 enzyme (1–2 mg, ~20 nmol) in 400 μl of Tris buffer (pH 7.5 for h15-LOX-1). The reaction was initiated by vigorous vortex mixing of the two phases. After 1.5 min, the hexane phase was collected and scanned from 200 to 350 nm using a PerkinElmer Lambda-35 spectrophotometer. Then the hexane phase was evaporated to about 2 ml under a stream of nitrogen, treated with ethanol (20 μl) and ethereal diazomethane for 10 s at 0°C, and then rapidly blown to dryness and kept in hexane at −80°C until further analysis.

### Reactions of fatty acids and fatty acid hydroperoxides with recombinant 15-LOX-1

For analysis of polar products, incubations with recombinant 15-LOX-1 and fatty acids or fatty acid hydroperoxides (10 or 20 μg/ml) were conducted at room temperature in UV cuvettes in 1 ml 50 mM Tris buffer (pH 7.5) containing 150 mM NaCl, with monitoring of the extent of the reaction by repetitive scanning (350–200 nm) in a Lambda 35 UV/Vis spectrometer (PerkinElmer). After 10–20 min and appearance of conjugated triene chromophores around 270 nm, reaction was halted by addition of 100 μl 1 M KH_2_PO_4_ buffer and 40 μl 1 N HCl to give approximately pH 3–4 and extracted using a 100 mg C18 Bond-Elut cartridge (Agilent), which was washed with water and eluted with methanol (MeOH).

### Mouse peritoneal resident macrophage incubations

Mouse peritoneal resident macrophages were collected as described (23) by lavage from naive mice (6–8 week old C57BL/6NCrl mice; Charles River Laboratories). All animal studies were approved and performed in accordance with guidelines provided by the Vanderbilt Medical Standing Committee on Animals.

After centrifugation at 400 *g* and addition of DMEM + 10% FBS, macrophages (5 × 10^6^ cells/ml) were incubated with zymosan A (200 μg/ml) and fatty acids or fatty acid hydroperoxides at 37°C for 30 min; the substrates were added in 2 μl ethanol per 0.5 ml cell incubation to give 20 μg/ml final concentration (equivalent to 60 μM 15*S*-HPEPE, 61 μM DHA, and 56 μM 17*S*-HPDHA). Incubations were stopped with two volumes of cold MeOH. After lysing with MeOH, the cell debris was removed by centrifugation at 10,000 rpm, the supernatant was mixed with three volumes of water, and the apparent pH was adjusted to approximately 3. The products were extracted on a 30 mg Oasis cartridge (Waters), eluted with MeOH, and analyzed by reversed-phase HPLC (RP-HPLC).

### HPLC analyses

From the biphasic reaction used for preparation of LTA-related epoxides, aliquots of the methylated hexane phase were analyzed by RP-HPLC using a Waters Symmetry column (25 × 0.46 cm), using a solvent of MeOH/20 mM triethylamine at pH 8.0 (90/10 by volume), at a flow rate of 1 ml/min, with on-line UV detection (Agilent 1100 series diode array detector with all 205, 220, 235, and 270 nm channels recorded at the same sensitivity). Further purification was achieved by SP-HPLC using a Beckmann Ultrasphere 5 μ silica column (25 × 0.46 cm) using a solvent of hexane/triethylamine (100/0.5) run at 1 ml/min. Aliquots of the room temperature incubation of recombinant human 15-LOX-1 with omega-3 fatty acid hydroperoxides were analyzed by RP-HPLC using a Waters Symmetry column (25 × 0.46 cm), using a solvent of acetonitrile (CH_3_CN)/H_2_O/HAc (45/55/0.01 by volume), at a flow rate of 1 ml/min. Aliquots of mouse macrophage incubations were analyzed by RP-HPLC using a Waters Symmetry column (15 × 0.21 cm), using a solvent of CH_3_CN/H_2_O/HAc (50/50/0.01 by volume), at a flow rate of 0.2 ml/min.

### LC-MS and GC-MS analyses

HPLC profiles were analyzed using a Thermo TSQ Vantage Triple Quadrapole MS instrument (Thermo Fisher Scientific, Waltham, MA). RP-HPLC analysis was performed with electrospray ionization in the negative ion mode. A Phenomenex Kinetex C18 2.6 μ column (100 × 3 mm) was eluted isocratically with CH_3_CN/water/glacial acetic acid (45:55:0.01 by volume) at a flow rate of 0.4 ml/min. The electrospray voltage was set at 4.0 kV: vaporizer temperature at 300°C; sheath and auxiliary gas pressure at 50 and 5 ψ, respectively; and capillary temperature at 300°C.

GC-MS was performed on hydroperoxy fatty acids by reduction with triphenylphosphine, SP-HPLC purification, methylation (diazomethane), hydrogenation (Pd/H_2_), silylation (*N*,O-bis(trimethylsilyl)trifluoroacetamide), and analysis on a Thermo-Finnigan DSQ mass spectrometer in the positive ion electron impact mode (70 eV).

### NMR analysis

^1^H NMR and ^1^H,^1^H COrrelated SpectroscopY NMR spectra were recorded on a Bruker AV-III 600 MHz spectrometer at 283 K. The parts/million values are reported relative to residual nondeuterated solvent (δ = 7.16 ppm for C_6_H_6_). Typically, 1,024 scans were acquired for a 1D spectrum on ~20 μg of LTA-related epoxide methyl ester.

## Results

### Preparation, purification, and characterization of LTA-related epoxides

**Fig. 1:**
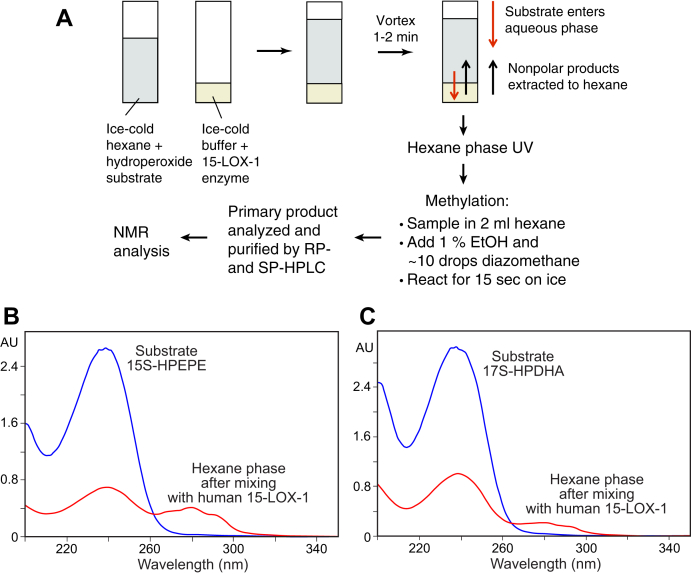
Preparation and isolation of LTA-related epoxides. A: Overview of the biphasic synthesis and simultaneous extraction methodology. B: UV spectra of the hexane phase of 15*S*-HPEPE reaction with 15-LOX-1 before and after mixing with the enzyme at 0°C. C: The UV profiles before and after reaction of 17S-HPDHA with 15-LOX-1. 15-LOX-1, 15-lipoxygenase-1; 15*S*-HPEPE, 15*S*-hydroperoxide of EPA; 17*S*-HPDHA, 17*S*-hydroperoxide-DHA; LTA, leukotriene A.

**Fig. 2:**
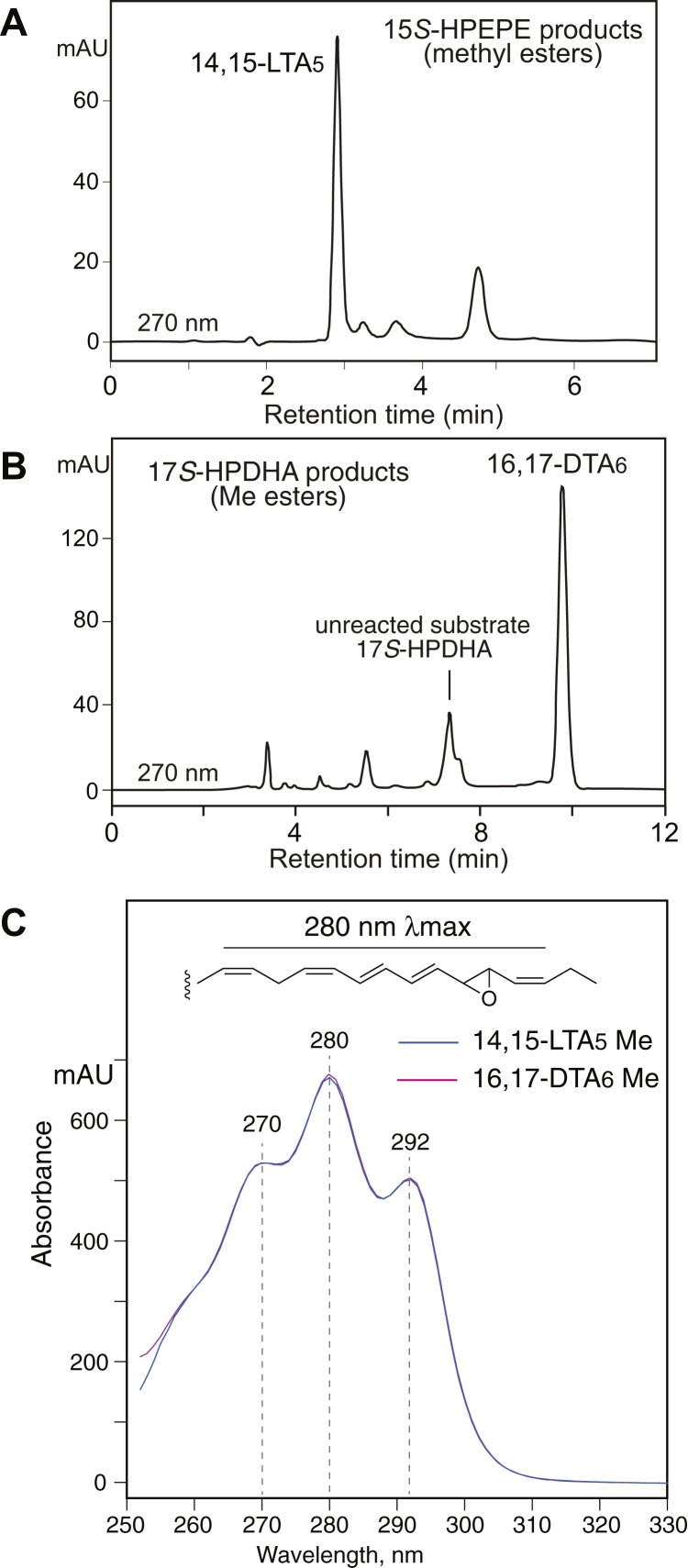
HPLC purification of LTA-related methyl esters. A: SP-HPLC analysis of the products of 15-LOX-1 with 15*S*-HPEPE using a silica guard column (0.46 × 4.5 cm), a flow rate of 0.5 ml/min, and a solvent system of hexane/triethylamine (100/0.5, by volume) with UV detection at 270 nm. B: RP-HPLC analysis of 15-LOX-1 products with 17*S*-HPDHA. RP-HPLC analysis used a Waters Symmetry C18 column (0.46 × 25 cm), a flow rate of 1 ml/min, and a solvent system of methanol/20 mM triethylamine at pH 8.0 (90/10, by volume) with UV detection at 270 nm. C: Overlay of the UV spectra of the two epoxides in hexane/0.5% triethylamine solvent. The spectra are essentially indistinguishable, as expected from their identical molecular environments (26), which includes the nonconjugated *cis* double bond separated by a methylene group on the carboxyl side of the *cis,trans,trans* conjugated triene, and the epoxide functionality and then a methylene and extra *cis* double bond toward the fatty acid tail as illustrated at the top of panel *C*. Spectra were recorded online on HPLC using an Agilent 1200 series diode array detector with hexane/triethylamine 100/0.5 by volume as solvent. 15-LOX-1, 15-lipoxygenase-1; 15*S*-HPEPE, 15*S*-hydroperoxide of EPA; 17*S*-HPDHA, 17*S*-hydroperoxide-DHA; LTA, leukotriene A; RP-HPLC, reversed-phase HPLC; SP-HPLC, straight-phase HPLC.

The methodology, outlined in Fig. 1A, was optimized from biphasic reaction conditions originally developed for isolation of fatty acid allene oxides (24, 25) and subsequently applied to LTA_4_ and its 5,6-*cis*-epoxy analogue (12). After vortex mixing of aqueous human 15-LOX-1 with a hexane solution of 15*S*-HPEPE or 17*S*-HPDHA at 0°C, UV spectroscopy of the hexane phase showed a decrease in substrate and appearance of a new chromophore with λmax at 280 nm characteristic of an LTA-type epoxide (Fig. 1B, C). Subsequent handling of the easily hydrolyzed epoxides was facilitated by prompt esterification to the methyl ester derivative using ethereal diazomethane and a trace of ethanol added to the hexane solvent. HPLC at room temperature with triethylamine in the running solvent allowed purification of the intact LTA-related methyl esters (see Materials and methods section and Fig. 2A–C).

**Fig. 3:**
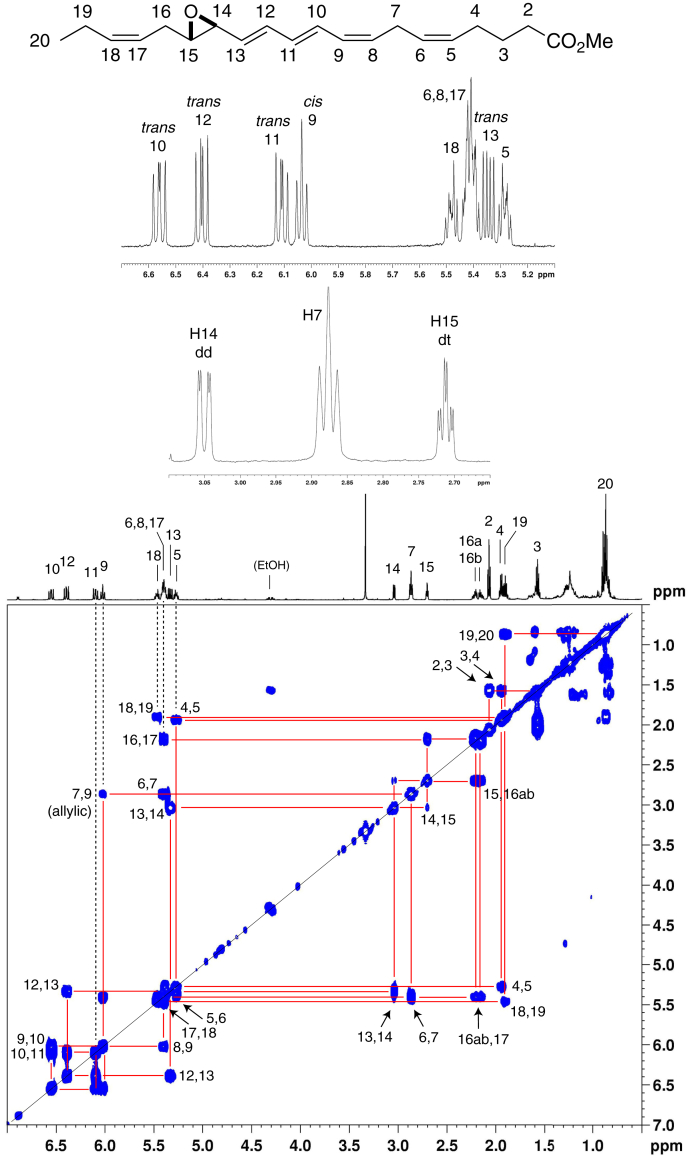
Proton NMR spectrum and COSY 2D spectrum of 14,15-LTA_5_ methyl ester. The COSY correlation spectrum allows assignment of the individual protons, and based on coupling constants, the expanded sections of the spectrum immediately below the structure establish the conjugated double bonds as 8*cis*,10*trans*,12*trans* and the epoxide configuration as 14,15-*trans*-epoxy (supplemental Table S1). 14,15-LTA_5_, 14*S*,15*S*-*trans*-epoxy-5*Z*,8*Z*,10*E*,12*E*,17*Z*-EPA; COSY, COrrelated SpectroscopY.

**Fig. 4:**
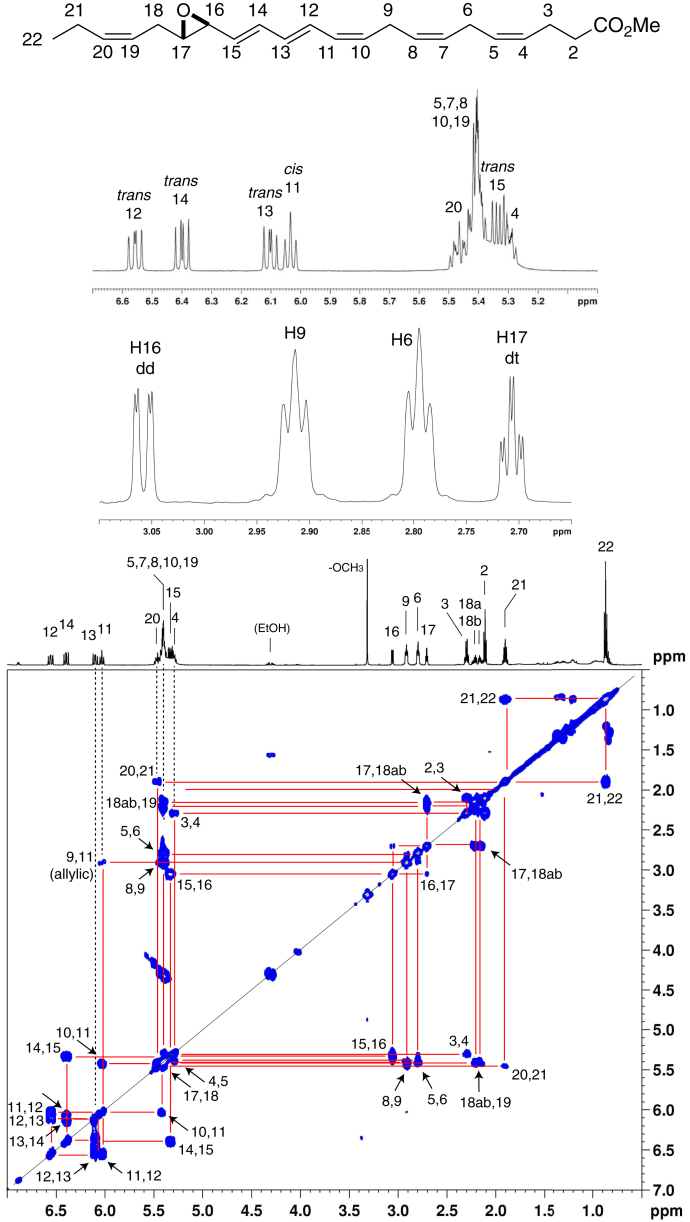
Proton NMR spectrum and COSY 2D spectrum of 16,17-DTA_6_ methyl ester. The COSY correlation spectrum allows assignment of the individual protons, and based on coupling constants, the expanded sections of the spectrum immediately below the structure establish the conjugated double bonds as 10*cis*,12*trans*,14*trans* and the epoxide configuration as 16,17-*trans*-epoxy (supplemental Table S2). 16,17-DTA_6_, 16,17-docosatriene A_6_ (analogue of LTA_4_), 16*S*,17*S*-*trans*-epoxy-4*Z*,7*Z*,10*Z*,12*E*,14*E*,19*Z*-DHA; COSY, COrrelated SpectroscopY.

Proton NMR with COrrelated SpectroscopY spectra of the epoxide methyl esters in C_6_D_6_ permitted direct assignment of the structures of the enzymatic products (Fig. 3, Fig. 4). Key features established through these analyses include the *cis*,*trans*,*trans* configuration of the conjugated triene, confirmed from the coupling constants of the 8,10,12 double bonds of 14*S*,15*S*-*trans*-epoxy-5*Z*,8*Z*,10*E*,12*E*,17*Z*-EPA (14,15-LTA_5_) (from EPA) and the 10,12,14 bonds of 16,17-docosatriene A_6_ (analogue of LTA_4_), 16*S*,17*S*-*trans*-epoxy-4*Z*,7*Z*,10*Z*,12*E*,14*E*,19*Z*-DHA (16,17-DTA_6_) (from DHA). The *trans*-epoxide configurations are clearly indicated by the 2 Hz coupling constants between the epoxide protons (Figs. 3, 4), with the full ^1^H-NMR spectra summarized in supplemental Tables S1 and S2. These data directly establish the structures of these 15-LOX-1-derived epoxides: 14,15-LTA_5_ as 14*S*,15*S*-*trans*-epoxy-5*Z*,8*Z*,10*E*,12*E*,17*Z*-EPA, and 16,17-DTA_6_ as 16*S*,17*S*-*trans*-epoxy-4*Z*,7*Z*,10*Z*,12*E*,14*E*,19*Z*-DHA, with the data for the latter in accord with the synthetic epoxide (13, 14).

### RP-HPLC analysis of products from recombinant 15-LOX-1 and 15*S*-HPEPE

The biphasic reactions described previously recover the nonpolar LTA-related epoxides in hexane, a solvent that will not efficiently extract more polar products such as dihydroxy derivatives. These more polar and stable products were prepared in a conventional incubation of 15*S*-HPEPE with 15-LOX-1 in pH 7.4 buffer at room temperature and extracted on an Oasis cartridge and analyzed by RP-HPLC with diode array UV detection; wavelength profiles were recorded at 205, 220, 235, and 270 nm along with full UV spectra on all chromatographic peaks.

**Fig. 5:**
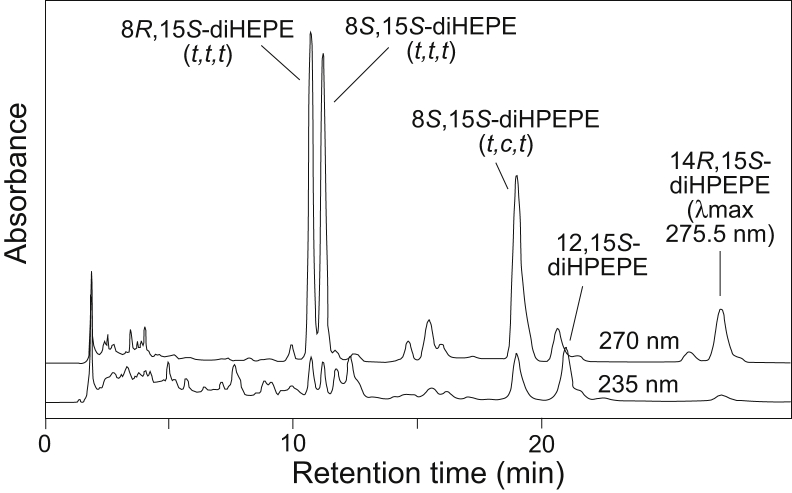
Reversed-phase HPLC analysis of the reaction of 15*S*-HPEPE with human 15-LOX-1. Column: Waters Symmetry C18, 25 × 0.46 cm; solvent, CH_3_CN/H_2_O/HAc (45/55/0.01, by volume); flow rate, 1 ml/min, UV detection at 270 and 235 nm. 15-LOX-1, 15-lipoxygenase-1; 15*S*-HPEPE, 15*S*-hydroperoxide of EPA.

Figure 5 shows the profiles at 235 and 270 nm and the associated product identifications. The two equal-sized peaks eluting at approximately 11 min in the 270 nm channel represent the C-8 diastereomers of 8,15*S*-dihydroxy-5*Z*,9*E*,11*E*,13*E*,17*Z*-EPA, the major hydrolysis products of 14,15-LTA_5_ with their characteristic all-*trans* conjugated triene chromophore (20). The other prominent product, eluting at 19 min, is an exact match in retention time and in its *trans*,*cis,trans* conjugated triene chromophore and 271 nm λmax with 8*S*,15*S*-dihydroperoxy-5*Z*,9*E*,11*Z*,13*E*,17*Z*-EPA prepared by double dioxygenation of EPA using soybean LOX-1 (cf. e.g., (27, 28)). In the CH_3_CN/water/acetic acid solvent system, hydroperoxy derivatives are well retained compared with their reduced hydroxy counterparts, and subsequent reanalysis of a reduced sample is associated with elution of the now-reduced products at shorter retention times (illustrated later for DHA dihydro(pero)xides).

Minor products were identified as 12,15*S*-dihydroperoxy-5*Z*,8*Z*,10*E*,13*E*,17*Z*-EPA (with an unusual UV spectrum with the same profile as a standard of the 20:4 analogue, depicted in the supplement of Ref. (29)), and identification confirmed by GC-MS of the triphenylphosphine-reduced hydrogenated methyl ester TMS ether derivative with the expected α-cleavage ions *m*/*z* 341 (C_1_–C_15_ minus TMSOH), 301 (C_1_–C_12_), 213 (C_12_–C_20_ minus TMSOH), and 173 (C_15_–C_20_); also, 14*R*,15*S*-dihydroperoxy-5*Z*,8*Z*,10*E*,13*E*,17*Z*-EPA, a double dioxygenation product with a conjugated triene chromophore with a relatively high λmax at 275.5 nm similar to the well-characterized 20:4 analogue (30, 31, 32). Eluting later than the products shown in Fig. 5 and with significant absorbance only in the 205 nm channel were two peaks at 28 and 30 min with the expected UV, chromatographic, and LC-MS (*m/z* 333, M-H) properties of 15-HPEPE-derived hepoxilins (epoxyalcohols). In fact, all the incubations in the current investigation with fatty acids and their hydroperoxides formed one or two relatively prominent hepoxilin-related peaks detected at 205 nm. These are likely to be enzymatic products, as well established with other LOXs reacting with fatty acid hydroperoxides (e.g., Refs. (33, 34, 35, 36)), although they were not the focus of the present study and not investigated further. Regarding the conjugated triene products, overall these results confirm the formation of 14,15-LTA_5_ by recombinant 15-LOX-1 in vitro along with double dioxygenation products as reported from arachidonic acid 15-hydroperoxide (e.g., Refs. (27, 31, 37)).

### HPLC-UV analysis of stable products from recombinant 15-LOX-1 and 17*S*-HPDHA

**Fig. 6:**
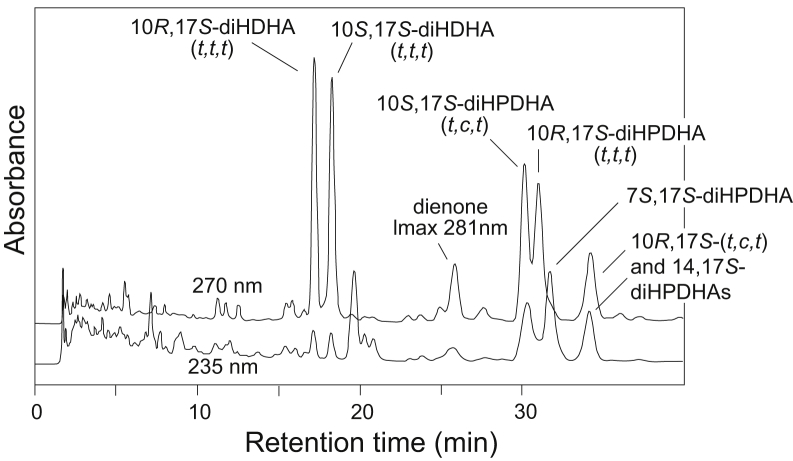
Reversed-phase HPLC analysis of the reaction of 17*S*-HPDHA with human 15-LOX-1. Column: Waters Symmetry C18, 25 × 0.46 cm; solvent, CH_3_CN/H_2_O/HAc (45/55/0.01, by volume); flow rate, 1 ml/min, UV detection at 270 and 235 nm. 15-LOX-1, 15-lipoxygenase-1; 17*S*-HPDHA, 17*S*-hydroperoxide-DHA.

**Fig. 7:**
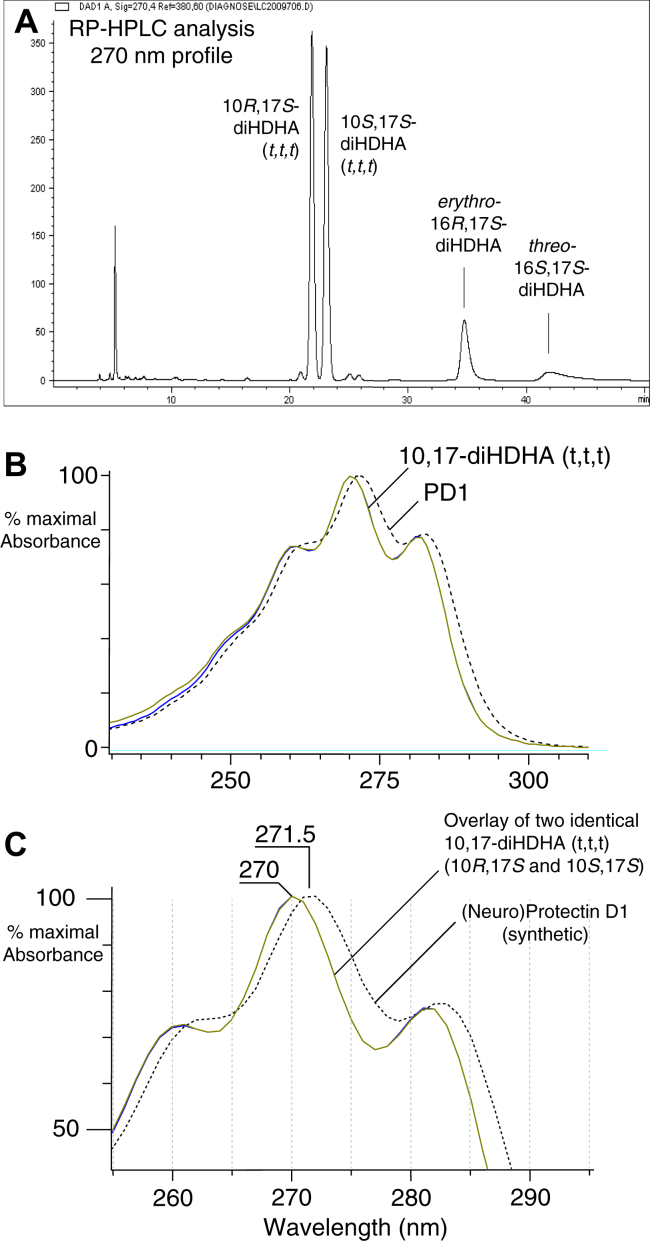
Detailed view of the UV spectra of the acid hydrolysis products of 16,17-DTA_6_. A: As shown previously for the synthetic epoxide methyl ester (13), acid hydrolysis gives four main products, separated here using a Waters Symmetry C18 column (25 × 0.46 cm) using a solvent system of acetonitrile/water/glacial acetic acid 60:40:0.01 at a flow rate of 0.5 ml/min with UV detection at 270 nm. B: UV spectra of the major hydrolysis products, the 10-17-diols (*t,t,t*), with the spectrum of synthetic protectin D1 added for comparison (dashed line). C: Detailed view of the spectra. The two 10,17-diols with all-*trans* conjugated triene and max at 270 nm have *identical* UV spectra (both are included here). The *erythro* and *threo* 16,17-diols have a λmax near 274 nm, although in this analysis there is a contaminating conjugated triene in the more prominent (*erythro*) peak as illustrated in supplemental Fig. S2. 16,17-DTA_6_, 16,17-docosatriene A_6_ (analogue of LTA_4_), 16*S*,17*S*-*trans*-epoxy-4*Z*,7*Z*,10*Z*,12*E*,14*E*,19*Z*-DHA.

There are parallels along with a few distinctive features in the 15-LOX-1 catalyzed transformation of the 17*S*-hydroperoxide of DHA compared with the 15*S*-hydroperoxide analogues of 20:4 and 20:5. Illustrated in Fig. 6 at retention times of 16–19 min, the significant formation of two diastereomers of 10,17*S*-diHDHA with their characteristic all-*trans* conjugated triene chromophore implicates formation of a 16,17-DTA_6_ epoxide. Their identities were confirmed by preparation of standards in two ways: by RP-HPLC and UV analysis of the acid hydrolysis of their epoxide precursor 16,17-DTA_6_ (Fig. 7) (with comparison to synthetic PD1 in supplemental Fig. S1) and by UV light and thiyl radical-induced isomerization of the soybean LOX-1 product 10*S*,17*S*-diHDHA (*t,c,t*) to the all-*trans* configuration (38, 39). The latter identified the second eluting peak of the pair as the 10*S*,17*S*-diHDHA (*t,t,t*) diastereomer, and therefore, the first eluting is assigned as 10*R*,17*S*-diHDHA (*t,t,t*). This order of elution is consistent with the arachidonate analogues in which 8*R*,15*S*-diHETE (*t,t,t*) elutes earlier than 8*S*,15*S*-diHETE (*t,t,t*) on RP-HPLC (30, 31). Further consideration of the acid hydrolysis products of 16,17-DTA_6_ methyl ester, and particularly the *erythro*-16,17-diol (which chromatographs on RP-HPLC as a mixture of *cis,trans,trans* and all-*trans* conjugated trienes), are given as supplemental data and supplemental Fig. S2.

**Fig. 8:**
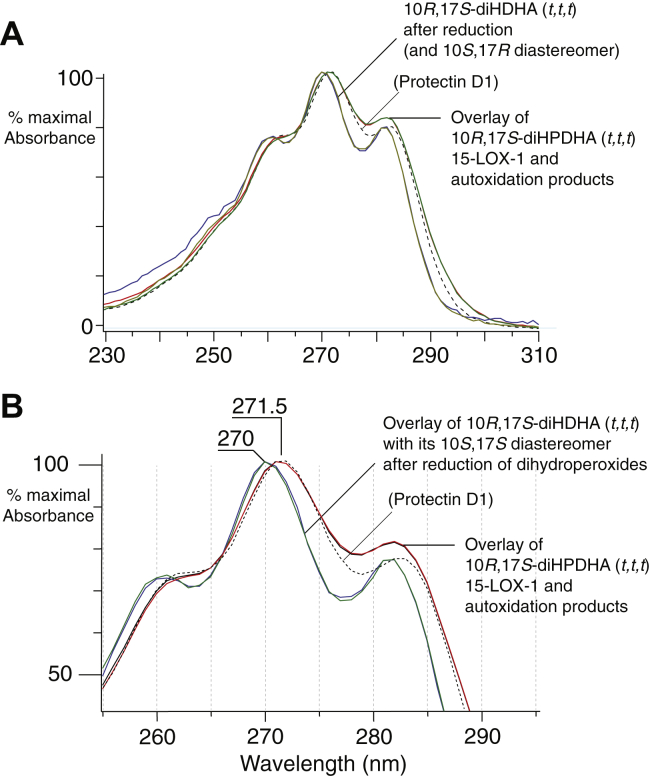
UV spectra of 10*R*,17*S*-diH(P)DHA (*t,t,t*) before and after reduction. A: Overlay of UV spectra of 10*R*,17*S*-diHPDHA (*t,t,t*) produced from 17*S*-HPDHA by 15-LOX-1 with the same dihydroperoxide produced by autoxidation of 17*S*-HPDHA (the latter chromatogram illustrated as supplemental Fig. S4). After reduction of the 15-LOX-1-derived products, the UV spectrum of 10*R*,17*S*-diHDHA (*t,t,t*) overlays perfectly with its 10*S*,17*S*-diHDHA (*t,t,t*) diastereomer. The spectrum of synthetic protection D1 is shown in dashed line for comparison. B: Detailed view of the UV spectra. Peak absorbance for the two dihydroxy isomers in the aliquot analyzed here were 12.5 and 6.3 mAU, respectively, attesting to reproducibility and sensitivity of the analysis. Spectra were recorded online on RP-HPLC in column solvent (CH_3_CN/H_2_O/HAc [45/55/0.01, by volume]). 15-LOX-1, 15-lipoxygenase-1; 17*S*-HPDHA, 17*S*-hydroperoxide-DHA; H(P)DHA (*t*,*t*,*t*) or (*t*,*c*,*t*), hydro(pero)xy-docosahexaenoic acid with conjugated triene *trans*,*trans*,*trans* or *trans*,*cis*,*trans*; RP-HPLC, reversed-phase HPLC.

The double dioxygenation products from the 17*S*-HPDHA/15-LOX-1 reaction eluted after 30 min and include 10*S*,17*S*-diHPDHA (*t,c,t*), 7*S*,17*S*-diHPDHA, and 10*R*,17*S*-diHPDHA (*t,t,t*) (Fig. 6). The first two are identical to the well-known soybean LOX-1 products from DHA (e.g., Refs. (27, 40, 41)). The 10*R*,17*S*-diHPDHA (*t,t,t*) dihydroperoxide is unusual in being formed in a chiral oxygenation with all-*trans* conjugation of the double bonds. Usually reactions with all-*trans* double bond conjugation are associated with racemic oxygenation, but in this case, only the 10*R*,17*S* (*t,t,t*) diastereomer is formed. The structural assignment was supported by *i*) analysis of NaBH_4_-reduced aliquots of the 15-LOX-1/17S-HPDHA reaction in which the two 10,17-diols with *identical* all-*trans* conjugated triene chromophores (16,17-DTA_6_ hydrolysis products) were now unequal in size—the first-eluting 10*R*,17*S* isomer being twice the size of its 10*S*,17*S* diastereomer because of the extra contribution from reduction of the 10*R*,17*S*-dihydroperoxide (supplemental Fig. S3), *ii*) the product matched the RP-HPLC retention characteristics of the 10*R*,17*S*-diHPDHA (*t,t,t*) produced by autoxidation of 17*S*-HPDHA (RP-HPLC of the autoxidation products shown in supplemental Fig. S4), and *iii*) the UV spectrum of 10*R*,17*S*-diHPDHA (*t,t,t*) from the 15-LOX-1/17*S*-HPDHA reaction exactly matched the spectrum of the autoxidation product, before and after reduction of the dihydroperoxide (Fig. 8).

The last UV peak on the Fig. 6 chromatogram is a mixture of 10*R*,17*S*-diHPDHA (*t,c,t*) and 14,17*S*-diHPDHA, the latter identified (in addition to its characteristic UV spectrum (29)) by GC-MS of the methyl ester TMS ether derivative after reduction and hydrogenation of the double bonds with prominent α-cleavage ions at *m*/*z* 173 (C_17_–C_22_), 213 (C_14_–C_22_ minus TMSOH), 329 (C_1_–C_14_), and 369 (C_1_–C_17_ minus TMSOH).

### RP-HPLC analysis of incubation of 15*S*-HPEPE with peritoneal resident mouse macrophages

Mouse resident peritoneal macrophages were isolated using the method adapted from a published protocol (23).The macrophage incubations were conducted with 15*S*-HPEPE DHA and 17*S*-HPDHA as substrate. Cellular inflammation is induced by zymosan A, or Ca-ionophore A23187 is used to enhance LOX activity.

**Fig. 9:**
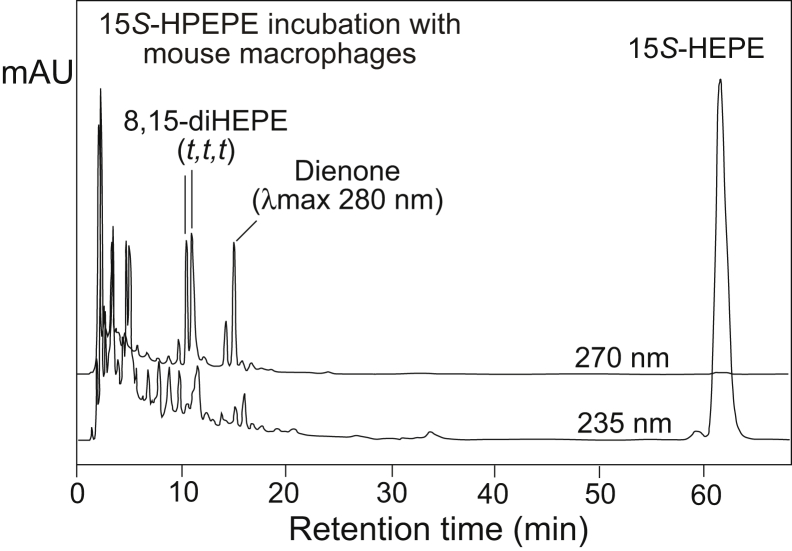
RP-HPLC analysis of the incubation of 15*S*-HPEPE with mouse resident peritoneal macrophages. In addition to dienone products that probably result from carbon chain cleavage of the substrate to C_15_ acid aldehydes, the nonenzymatic hydrolysis products, 8,15-diHEPEs (*t,t,t*), were detected, indicating the production of 14,15-LTA_5_. 14,15-LTA_5,_ 14*S*,15*S*-*trans*-epoxy-5*Z*,8*Z*,10*E*,12*E*,17*Z*-EPA; 15*S*-HPEPE, 15*S*-hydroperoxide of EPA; RP-HPLC, reversed-phase HPLC.

Figure 9 demonstrates the products derived from the incubation of the EPA-derived hydroperoxide 15*S*-HPEPE with mouse macrophages. Two equal-sized peaks of 8,15-diHEPEs with identical all-*trans* conjugated triene chromophores elute at ~11 min retention time, indicating the production of 14,15-LTA_5_. At 14 and 15 min retention time are two products that were not evident in the in vitro incubations with recombinant 15-LOX-1; each exhibits the smooth UV profile of a conjugated dienone chromophore with λmax values at 278 and 280 nm, respectively. Relatively polar products with conjugated dienone UV spectra are produced by chain cleavage of 15-HPEPE, and a tentative assignment for the two dienones would be 15-oxo-pentadecenoic acids with *EE* and *ZE* double bonds in conjugation with the C15 aldehyde. Eluting last in Fig. 9 is the peak of 15*S*-HEPE, reduced to the hydroxy form by cellular peroxidases.

### RP-HPLC analysis of DHA and 17*S*-HPDHA metabolism in peritoneal resident mouse macrophages

**Fig. 10:**
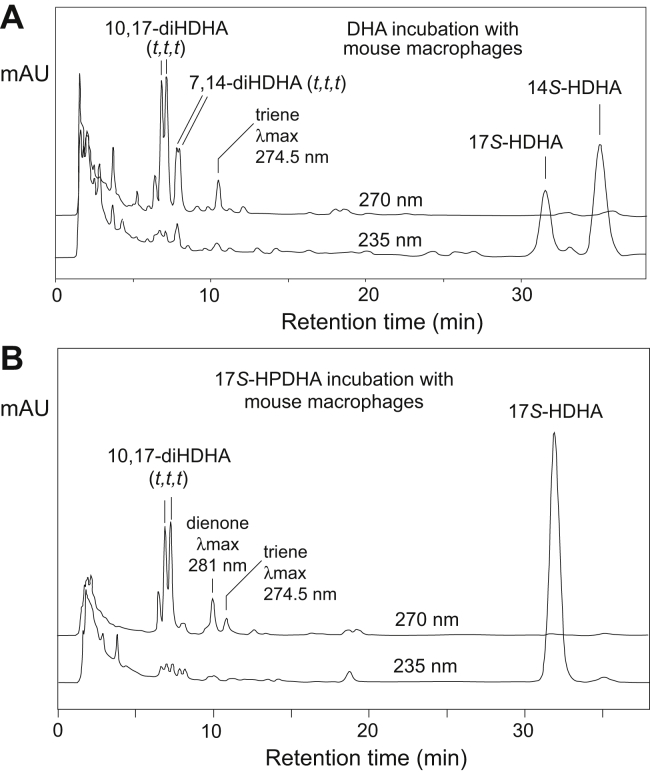
RP-HPLC analysis of the incubation of (A) DHA and (B) 17*S*-HPDHA with mouse resident peritoneal macrophages. Column: Waters Symmetry C18, 15 × 0.21 cm; solvent, CH_3_CN/H_2_O/HAc (50/50/0.01, by volume); flow rate, 0.2 ml/min, UV detection at 270 and 235 nm. Protectin D1 was not detected; its retention time in this system is very short before or merged with 10*S*,17*S*-diHDHA (*t,t,t*), the second hydrolysis peak of the LTA-related epoxide 16,17-DTA_6_ (cf. supplemental Fig. S1). 16,17-DTA_6_, 16,17-docosatriene A_6_ (analogue of LTA_4_), 16*S*,17*S*-*trans*-epoxy-4*Z*,7*Z*,10*Z*,12*E*,14*E*,19*Z*-DHA; 17*S*-HPDHA, 17*S*-hydroperoxide-DHA; LTA, leukotriene A; RP-HPLC, reversed-phase HPLC.

Incubation of DHA with mouse macrophages led to the formation of mono-oxygenation products 14*S*-HPDHA and 17*S*-HPDHA, which, because of the presence of cellular peroxidases, appeared as their reduced hydroxy counterparts 14*S*-HDHA and 17*S*-HDHA (Fig. 10A). (UV spectra of the monohydroperoxides or of dihydroperoxides differ from their hydroxy counterparts, supplemental Fig. S5). Different from the enzymatic incubations in which double dioxygenation products are detected prominently, the macrophage incubations mainly converted 17*S*-HPDHA and 14*S*-HPDHA to LTA-type epoxides, which were manifested by the production of their nonenzymatic hydrolysis products, a pair of 10,17-diHDHAs followed by a smaller pair of 7,14-diHDHAs, each with their characteristic all-*trans* conjugated triene chromophore. Incubations with 17*S*-HPDHA and 14*S*-HPDHA (data not shown) confirmed the secondary transformations in the pathway. It is noted that mouse macrophages transformed DHA to 14*S*-HPDHA and 17*S*-HPDHA in a ratio of ~2:1, whereas the secondary transformations to LTA epoxides are more efficient for 17*S*-HPDHA than for 14*S*-HPDHA. This is compatible with the ability of mouse 12/15-LOX to readily initiate reaction by hydrogen abstraction from the bisallylic position at C12, with much lower probability of hydrogen abstraction further up the carbon chain at the next available methylene at C9, the latter required for transformation of 14-HPDHA to a 13,14-DTA_6_-related maresin epoxide.

A similar, relatively simple, profile of diHDHAs was produced in incubations of 17*S*-HPDHA with mouse peritoneal macrophages (Fig. 10B). The main products with conjugated double bonds were the two 16,17-DTA_6_ hydrolysis products, the 10,17-diHDHAs with all-*trans* conjugated triene chromophores. The bioactive endproducts, PD1 derived from 16,17-LTA_6_ and maresin 1 from 13,14-LTA_6_, with their unique *trans,trans,cis*-conjugated triene configuration were not detected in the macrophage incubations. One explanation for the failure in detecting PD1 is that mouse macrophages do not possess the specific LTA hydrolase. On the other hand, maresin 1 was detected from mouse macrophages in the work leading to its discovery (18). We can only state that the incubation conditions utilized here were not associated with any detectable formation of PD1 or MaR1, even when the “baseline” UV peaks were examined closely.

## Discussion

### Utility of the biphasic system for LTA-related epoxide biosynthesis

This merits comment. *1*) Producing efficient conversion to product in a very short time is critical, and given the potential instability of the epoxide, the LOX enzyme needs to be highly active at 0°C. *2*) The pH of the aqueous phase of the biphasic system needs to be optimized, which often requires a balance between the enzymatic activity, the back-extraction efficiency, and the stability of the resulting epoxide in the aqueous phase. It helps a great deal that the epoxide product is even less polar than the hydroperoxide substrate, optimizing its partitioning and recovery in the hexane. The nonpolarity of the epoxide is also associated with a higher pKa (likely higher than pH 7), again allowing for its accumulation in the hexane phase while allowing an optimal pH for enzyme activity. *3*) We kept the reacting environment anaerobic, which hinders oxygenation reactions and may favor the dehydration reaction to the LTA-related epoxide. *4*) Hexane as the organic phase will not impair most enzymes as the solvent itself is so nonpolar that it is essentially occluded from an aqueous environment, whereas the nonpolarity of hexane preserves the accumulating epoxide. With several parameters helping recovery of the epoxide, and some hurting (low temperature, extraction at pH >7, short reaction time), the yield of epoxide is only 5–10%, yet sufficient to achieve characterization by proton NMR spectroscopy, matching the quality of LTA_4_ itself (12) and the “16*S*,17*S*-epoxyprotectin” prepared by total chemical synthesis (13).

### Nomenclature of the epoxides

Currently, there is no set name for the EPA- and DHA-derived LTA-related epoxides. 14,15-LTA_5_ is appropriate for the EPA analogue (cf. (42)). For the DHA/17*S*-HPDHA-derived epoxide, it has been referred to as a 16,17-epoxide-containing intermediate (17), as 16*S*,17*S*-epoxyprotectin (13), DHA-derived protectin-related epoxide (15), and (by analogy to the 12-LOX analogue) 16*S*,17*S*-epoxy-DHA (43). The name “leukotriene A_6_” is inappropriate for several reasons. The name 16,17-DTA_6_ is based on the original description of the group of products by Serhan *et al.* (44) as docosatrienes, which seems apt and well put.

### Product analyses and identifications

**Scheme 2:**
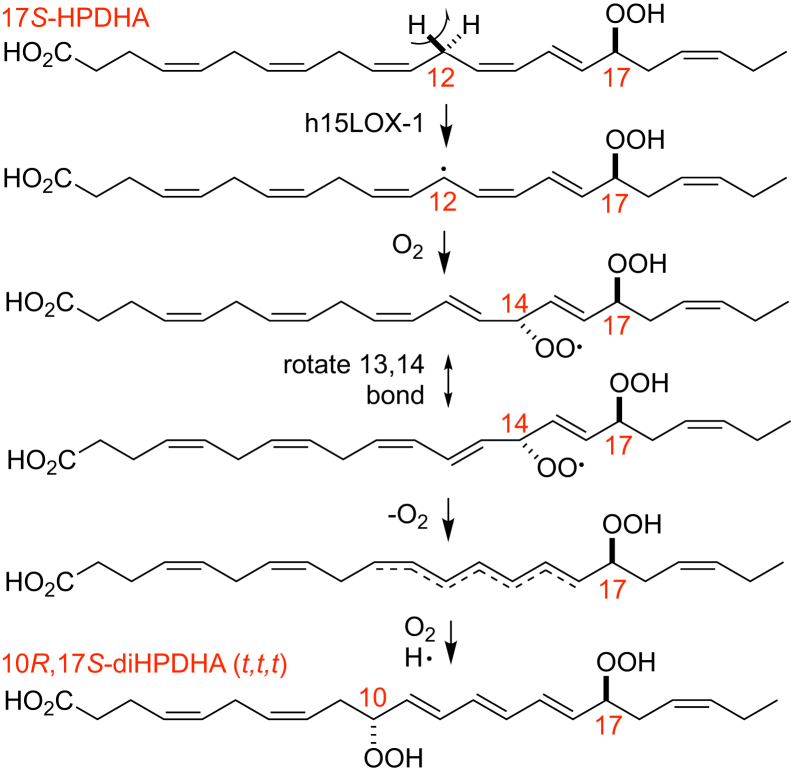
Proposed mechanism of 10*R*,17*S*-diHPDHA (*t,t,t*) synthesis.

In the recombinant 15-LOX-1 reactions, the products mainly were dihydroxy derivatives from hydrolysis of LTA-related epoxides and dihydroperoxides from double dioxygenations. Among the 17*S*-HPDHA/15-LOX-1 products, 10*R*,17*S*-diHPDHA (*t,t,t*) is of interest in being a chiral hydroperoxide with all-*trans* configuration of the double bonds involved and formed in the absence of the corresponding 10*S*,17*S*-diastereomer. To the best of our knowledge, this transformation is unique in the literature on LOX biochemistry. All other oxygenations with all-*trans* conjugation of the double bonds, including to monohydroperoxides, are racemic (cf. (45)). As in autoxidation, the mechanism of formation of the 10*R*,17*S*-dihydroperoxide with all-*trans* conjugated double bonds may involve the on-off intermediacy of molecular oxygen as a peroxyl radical, in this case at C14, allowing swiveling of the carbon chain into a *trans* configuration, with subsequent β-scission of the 14-peroxyl radical and reoxygenation and formation of a 10-hydroperoxide, fixing the unsaturated carbon chain as all-*trans* (Scheme 2) (cf. Ref. (45)). Notably, 10*R*,17*S*-diHDHA (*t,t,t*) (the hydroxy analogue) exhibits similar biological activity to 10*S*,17*S*-diHDHA (*t,c,t*) in inhibiting neutrophil migration in an in vivo mouse model (17). Nonetheless, there was none detectable in mouse macrophages incubated with DHA or 17*S*-HPDHA; if it had been produced, it would be reduced in cells and detected as a heightened HPLC-UV peak of the first 10,17-diol formed from the epoxide 16,17-DTA_6_—and that clearly was not the case (cf. Fig. 10 with supplemental Fig. S2). A second issue of special interest is the absence of PD1 (defined as 10*R*,17*S*-diHDHA (*t*,*t*,*c*)) as a detectable product from mouse macrophages, to be discussed further.

### UV spectra as a criterion for product identification

Our study re-establishes UV spectroscopy, as originally demonstrated for the leukotrienes (20, 21), as affording distinctive attributes for the identification of the various LOX-derived resolvins and protectins. Why is this methodology not used to its full extent in practice? The available instrument might be a factor, although we have used a commonly available commercial HPLC diode array detector (Agilent 1100 and 1200 series). While the accuracy and spectral resolution can be instrument dependent, the within-instrument reproducibility with a diode array detector (no moving parts) is excellent: we estimate the precision of spectra (reproducibility within our instruments) to be better than ±0.1 nm, standard deviation. Of course, the requirement for a quality UV spectrum relies heavily of having a quality and directly comparable background or reference scan or scans; this might be hard to achieve when gradient elution is constantly changing the solvent mixture; all our analyses used isocratic elution. Obviously, there should be minimal interference from coeluting compounds, which again might not be achieved on short runs with strong gradient elution on HPLC. Different instruments afford different spectral resolution, so extremely subtle differences require direct comparison on the same instrument. That said, for the most part, the UV spectra of resolvins and protectins on one instrument closely match that on another and are recognizable as such in publications (cf. (46, 47)).

### Evidence for PD1 formation by biosynthesis is not conclusive

Although detection of PD1 (aka neuroprotectin D1 [NPD1]) first appeared in 2004 (48, 49) and included analyses proposing a mechanism of biosynthesis and chemical structure (50), the very existence of PD1 (same as NPD1) as a significant biosynthetic product is not achieved with the level of certainty afforded to LTB_4_. The low amounts deduced of PD1 biosynthesis, which may never have exceeded a few nanograms, have compromised proof of the deduced chemical structure. From biological samples, there are no clean UV spectra of PD1 of the quality well established for LTB_4_ (20), and as noted before (46), the early published spectra from biological sources more closely resemble the double oxygenation product 10*S*,17*S*-diHPDHA with a *trans,cis,trans* conjugated triene chromophore (cf. Ref. (50) with (41, 46, 47, 51)), or have a different profile altogether from the latter and authentic PD1 (52). Although there are excellent mass spectral analyses on the synthetic molecules (53), on account of the low levels in biological samples, the reported mass spectra attributed to PD1 are often weak and compromised by interfering ions and are not distinguished from other potential 10,17-diol isomers. Indeed, it is a matter of record that LTB_4_ was never distinguished from its 5,12-dihydroxy isomers based on a mass spectrum. Its identification relies on distinctive HPLC retention times (reversed phase and normal phase) along with its characteristic UV spectrum. The same would apply to the LTB_4_ analogues PD1 (and MaR1), albeit not attained to date on a biosynthetic product. No enzyme is reported that can form specifically PD1 from 16,17-DTA_6_, and the reported biological transformation of 16,17-DTA_6_ to PD1 in cells is lacking in the features expected by parallels to the conversion of synthetic LTA_4_ to LTB_4_, namely no discernible indication of yield, and on the published HPLC chromatogram (13), lack of any nonenzymatically derived 10,17-diols formed from the 200 ng 16,17-DTA_6_ administered to cells (13) (as expected, and illustrated before with cellular conversion of LTA_4_ to LTB_4_ (54, 55)). As the evidence stands currently, further work is required to substantiate or refute the existence of PD1 as a natural product.

### Biological activity of resolvins and docosatrienes

The potent biological activities of the resolvins and protectins is impressive and strongly supported and extended by the availability of synthetic isomers and analogues. Many outstanding studies document the proresolving activities of these molecules and help establish structure-activity relationships. Often, among the docosatrienes, PD1 is among the most potent (56, 57, 58), although its positional isomers are active too, and in some instances, a non-natural isomer is the most potent (17). Altogether the evidence indicates that several of the LOX-derived EPA and DHA diols are biologically active (17, 59, 60). Indeed, the original reports of pharmacological studies with PD1 (as “NPD1”) were conducted with biosynthetic material that most likely was 10*S*,17*S*-diHDHA with a *trans,cis,trans* conjugated triene chromophore or some other isomer that can be prepared by biosynthesis (and therefore not the structure defined as PD1 or NPD1) (cf. Refs. (41, 46, 47, 51)). The evidence suggests that each of the derivatives we characterize in this study (several of which are already described) merit consideration as potential bioactive molecules.

## Data availability

Data are contained in the article and supplemental data.

## Supplemental data

This article contains supplemental data.

## Conflict of interest

A. R. B. is a consultant for Lonza Pharma and Biotech. All other authors declare that they have no conflicts of interest with the contents of this article.
