# Supplementary material for: Analysis of 12/15-lipoxygenase metabolism of EPA and DHA with special attention to authentication of docosatrienes
Source: J Lipid Res. 2021 May 20;62:100088. doi: 10.1016/j.jlr.2021.100088 (PMC8219989; doi:10.1016/j.jlr.2021.100088)
Supplement: Supplemental Figures S1–S5 and Tables S1–S2 [file mmc1.pdf]

Supplementary data for:

Analysis of 12/15-lipoxygenase metabolism of EPA and DHA with special attention to authentication of docosatrienes

Jing Jin, William E. Boeglin, and Alan R. Brash[1]

Department of Pharmacology and the Vanderbilt Institute of Chemical Biology, Vanderbilt University, Nashville, TN 37232

[1] Corresponding author: alan.brash@vanderbilt.edu

Supplemental Figure S1:
RP-HPLC of 10*R*,17*S*- and 10*S*,17*S*-diHDHA (*t,t,t*) and synthetic Protectin D1

Supplemental Figure S2:
Detailed view of the UV spectra of the acid hydrolysis products of 16,17-DTA$_6$ methyl ester

Supplemental Figure S3:
RP-HPLC analysis of the products of 17*S*-HPDHA reaction with 15-LOX-1, before and after reduction with sodium borohydride

Supplemental Figure S4:
RP-HPLC profile of 10,17-diHPDHAs from autoxidation of 17*S*-HPDHA

Supplemental Figure S5:
UV Spectra of 17-Hydro(pero)xy-DHA and 10*S*,17*S*-diHydro(pero)xy-DHA (*t,c,t*)

Supplemental Table S1: **^{1}H-NMR (600 MHz) in C_6D_6 of 14*S*,15*S*-epoxy-eicosapenta-5*Z*,8*Z*,10*E*,12*E*,17*Z*-enoate methyl ester (14,15-Leukotriene A$_5$ methyl ester)**

Supplemental Table S2: **^{1}H-NMR (600 MHz) in C_6D_6 of 16*S*,17*S*-epoxy-docosahexa-4*Z*,7*Z*,10*Z*,12*E*,14*E*,19*Z*-enoate methyl ester (16,17-Docosatriene A$_6$ methyl ester)**

Supplemental Figure S1:

RP-HPLC of 10*R*,17*S*- and 10*S*,17*S*-diHDHA (*t,t,t*) and synthetic Protectin D1

Comparison of mixtures run using a Waters Symmetry 5 μ C18 column (25 x 0.46 cm) at flow rate of 1 ml/min.

A: Isocratic elution with a solvent of MeOH:H_2O:HAc (70:30:0.01, by volume)

B: Isocratic elution with a solvent of CH_3CN:H_2O:HAc (45:55:0.01, by volume). On another Waters Symmetry column with the same solvent system, PD1 eluted as a distinct peak between the two all-*trans* 10,17-diols (nearer the second all-*trans* isomer).

C: Overlay of their UV spectra

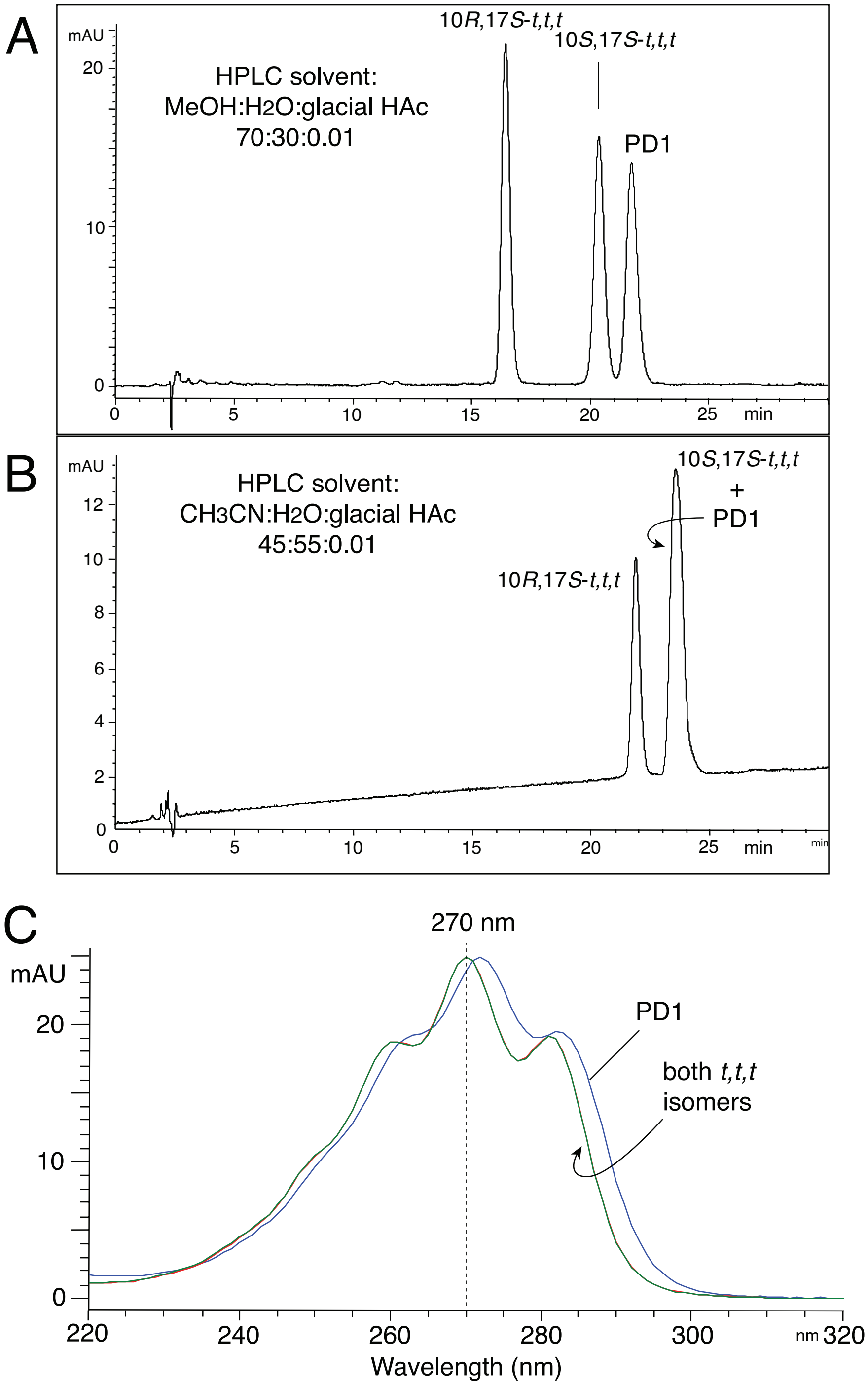

Supplemental Figure S2

Detailed view of the UV spectra of the acid hydrolysis products of 16,17-DTA$_6$ methyl ester

A: (Same as main Figure 7A): acid hydrolysis products of the LTA-related epoxide 16,17-DTA$_6$ methyl ester, separated using a Waters Symmetry C18 column (25 x 0.46 cm) using a solvent system of acetonitrile/water/glacial acetic acid 60:40:0.01 at a flow rate of 0.5 ml/min with UV detection at 270 nm.

B: Expanded view of the same HPLC peaks of the first major 10,17-diol (left side) and the first eluting 16,17-diol (right side)

C: Detailed view of the UV spectra, showing overlay of four scans across the HPLC peaks with identical spectra of the 10,17-all-*trans* diol, and indicting the presence of a contaminating conjugated triene in the HPLC peak of the *erythro*-16,17-diol. In another hydrolysis reaction of the same preparation of 16,17-DTA$_6$ methyl ester there was less evidence of the contaminating conjugated triene in the RP-HPLC peak of the *erythro*-16,17-diol, yet further by SP-HPLC analysis showed the presence of two products (panel D – **next page**).

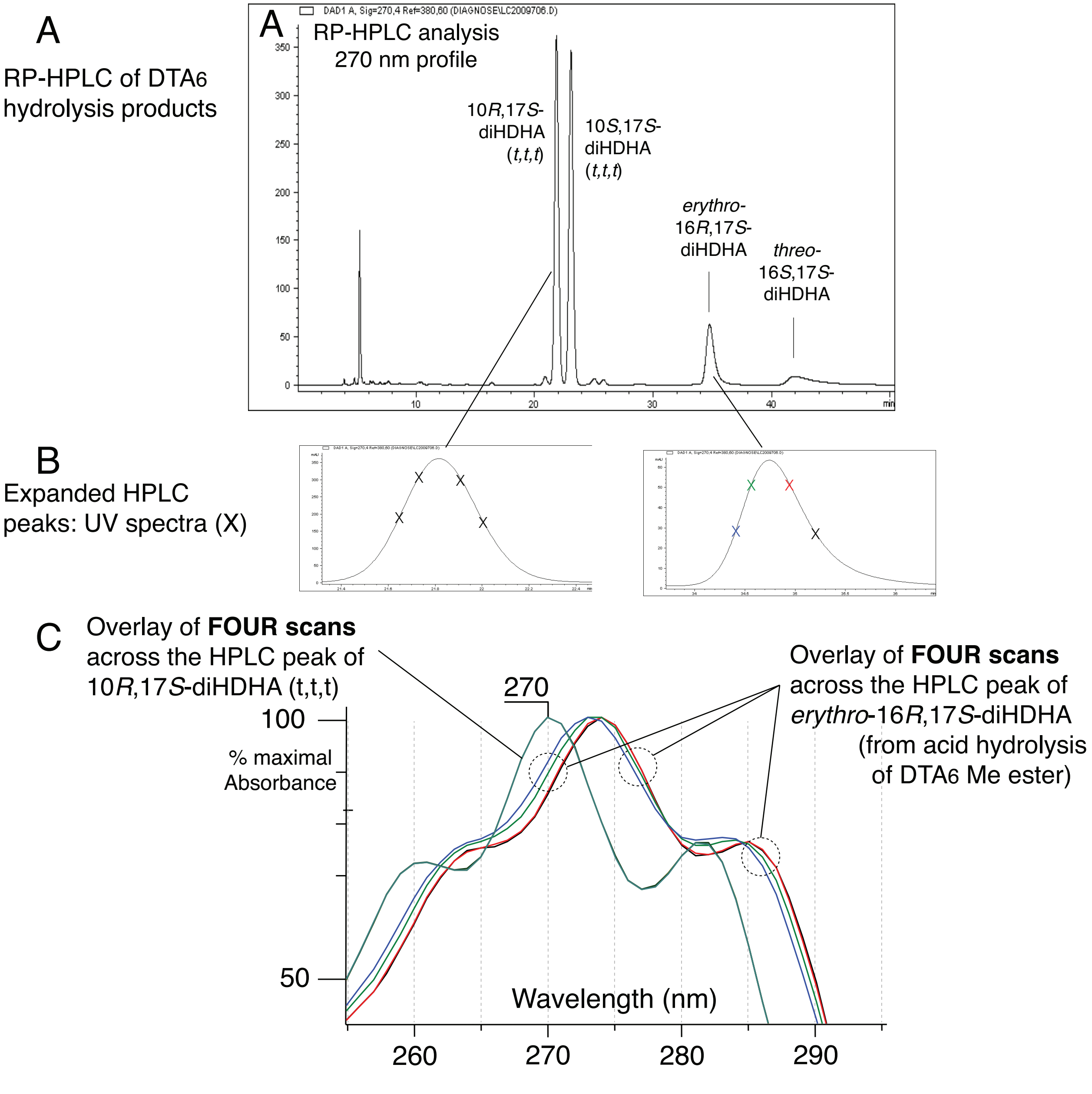

Supplemental Figure S2 (continued)

D: SP-HPLC of the *erythro*-16,17-diol methyl ester collected from RP-HPLC showing the presence of two components, the first and major peak with λmax at 274 nm in SP solvent (16,17-*erythro*-diol with *cis,trans,trans* conjugated triene) and minor with λmax 270 nm (16,17-diol with all-*trans* triene); *inset*: UV spectra. Column: Phenomenex Luna 5 μ silica (25 x 0.2 cm) with a solvent of hexane/isopropanol/glacial acetic acid (100:5:0.1, by volume) at a flow rate of 0.4 ml/min.

D

SP-HPLC of *erythro*-16,17-diol Me ester from RP-HPLC

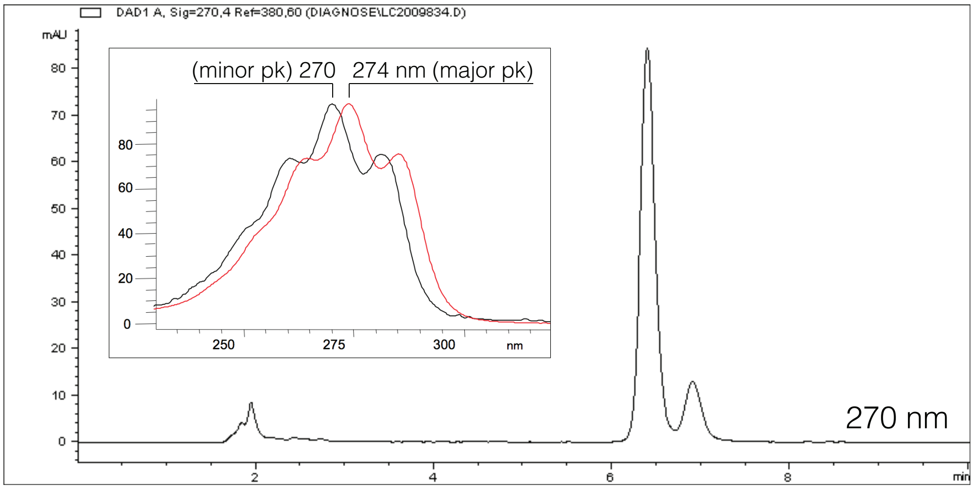

Supplemental Figure S3

RP-HPLC analysis of the products of 17*S*-HPDHA reaction with 15-LOX-1, before and after reduction with sodium borohydride

A: Before reduction, in this solvent the dihydroxy products elute earlier than the dihydroperoxides; at 18 – 20 min are two 10,17-dihydroxy diastereomers with all-*trans* conjugated triene formed by hydrolysis of the LTA-related epoxide (16,17-DTA_6), and at 32 – 35 min two 10,17-dihydroperoxides,10*S*,17*S*-diHPDHA (*t,c,t*) and 10*R*,17*S*-diHPDHA (*t,t,t*).

B: After reduction, the peak of the first-eluting dihydroxy (10*R*,10*S*) isomer doubles in size due to contribution from reduction of the 10*R*,10*S* dihydroperoxide (indicated with arrow).

Aliquots of the reaction were analyzed before and after $NaBH_4$ treatment using a Waters Symmetry C18 column 25 x 0.46 cm) with a solvent of acetonitrile/water/glacial acid 45:55:0.01 (by volume), at a flow rate of 1 ml/min, showing the UV profile at 270 nm.

RP-HPLC, 270 nm, 17*S*-HPDHA product profile with 15-LOX-1

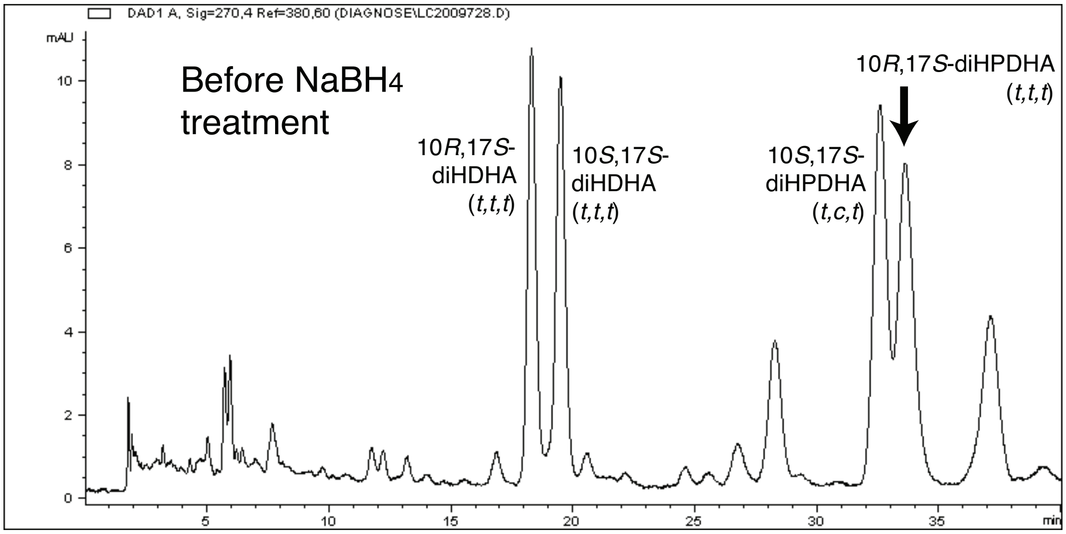

RP-HPLC, 270 nm, 17*S*-HPDHA product profile with 15-LOX-1 after NaBH4 reduction

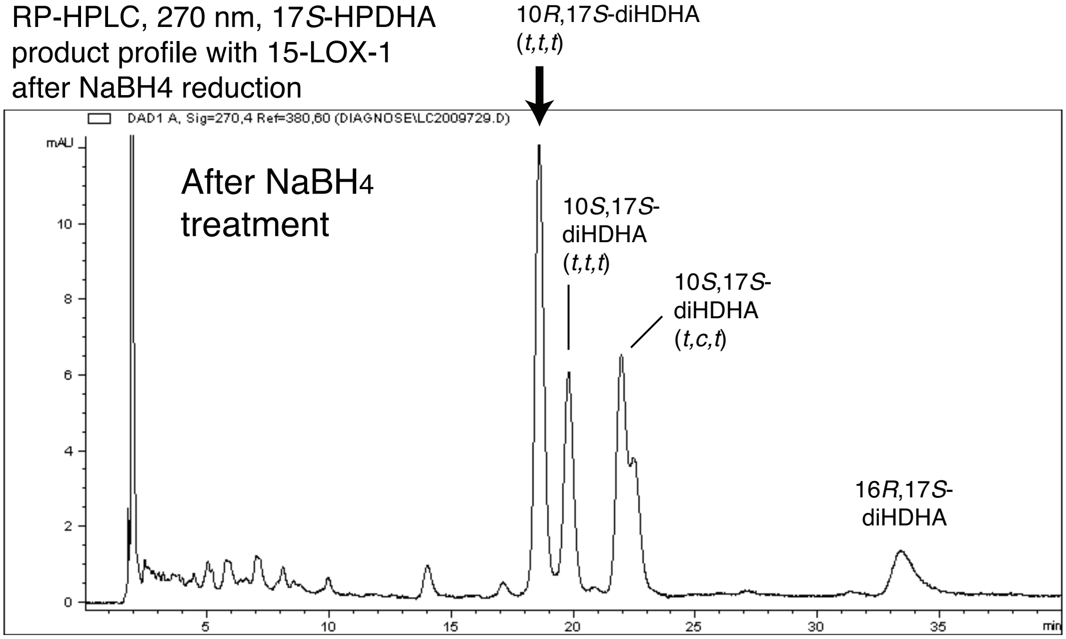

Retention time (min)

Supplemental Figure S4

RP-HPLC profile of 10,17-diHPDHAs from autoxidation of 17*S*-HPDHA

The partial chromatogram shows elution of the 10,17-diHPDHAs detected at 270 nm. The first eluting product at 30.5 min is identical in retention time and UV spectrum to 10*S*,17*S*-diHPDHA (*t,c,t*), the double dioxygenation product of soybean lipoxygenase. Its 10*R*,17*S*-diHPDHA (*t,c,t*) diastereomer of equal abundance elutes at 34.8 min. The pair of smaller 10,17-diHPDHAs (*t,t,t*) at 31.5 and 36.9 min are assigned as 10*R*,17*S* and 10*S*,17*S* in order of elution based on the precedent with 8,15-diH(P)ETE analogues from arachidonic acid (refs 29 & 30 in main text). The RP-HPLC was run using a Waters Symmetry C18 column (25 x 0.46 cm) with a solvent of acetonitrile/water/glacial acid 45:55:0.01 (by volume), at a flow rate of 1 ml/min, showing the UV profile at 270 nm.

Autoxidation of 17*S*-HPDHA: RP-HPLC of 10,17-dihydroperoxides

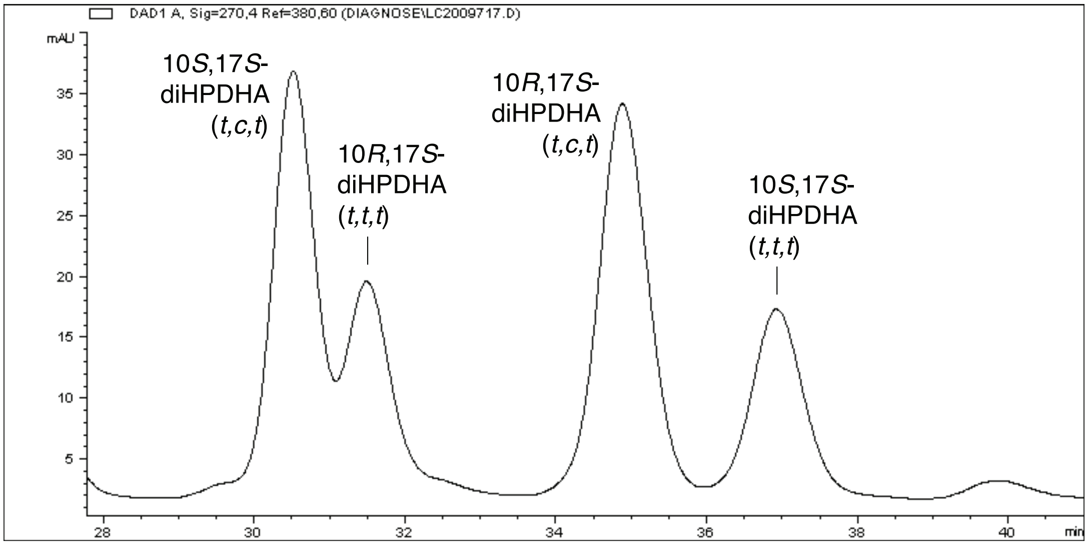

Supplemental Figure S5

UV Spectra of 17-Hydro(pero)xy-DHA and 10*S*,17*S*-diHydro(pero)xy-DHA (*t,c,t*)

17-Hydroxy and 17-Hydroperoxy-DHA cannot be distinguished reliably based on λmax (237.5 and 238 nm, respectively), but the hydroperoxide chromophore extends significantly towards higher wavelengths. The double deoxygenation product 10*S*,17*S*-diHPDHA is readily distinguished from its reduction product, 10*S*,17*S*-diHDHA. The UV spectrum of synthetic Protectin D1 (dashed line) is included for comparison.

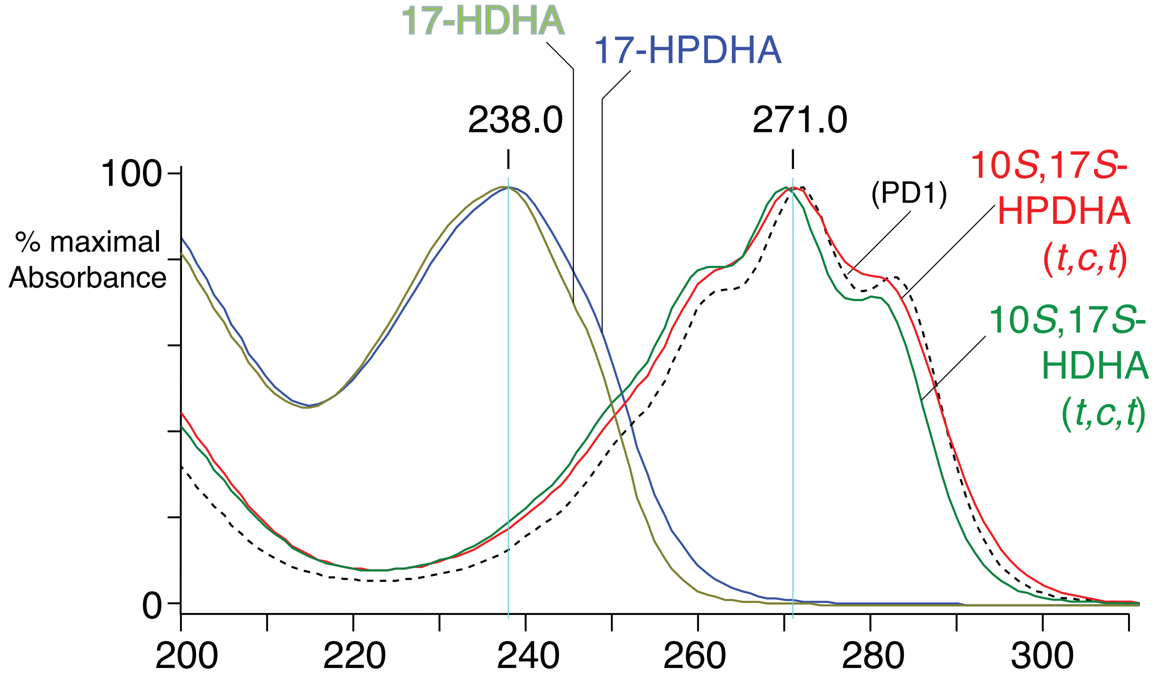

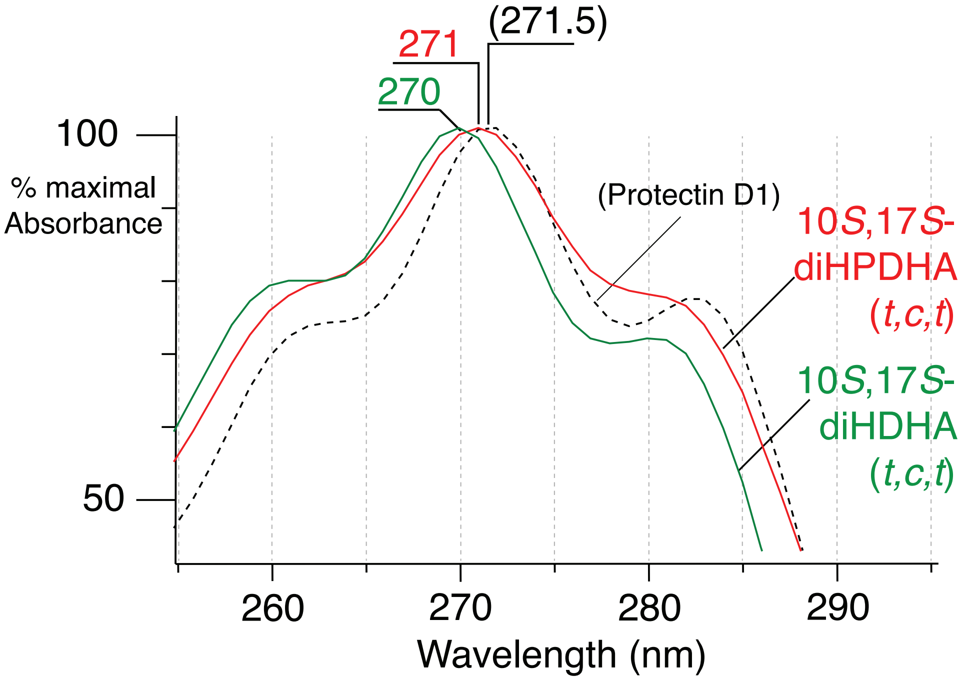

Supplemental Table S1: ^{1}H-NMR (600 MHz) in C_6D_6 of 14*S*,15*S*-epoxy-eicosapenta-5*Z*,8*Z*,10*E*,12*E*,17*Z*-enoate methyl ester (14,15-Leukotriene A_5 methyl ester)

Chemical shift (ppm)	Proton(s)	Number of protons	Multiplicity	Coupling Constants (Hz)
6.56	10	1	dd	$J_{9,10}$ = 11.4, $J_{10,11}$ = 14.9
6.405	12	1	dd	$J_{11,12}$ = 10.9, $J_{12,13}$ = 15.2
6.11	11	1	dd	$J_{10,11}$ = 14.9, $J_{11,12}$ = 10.9
6.035	9	1	t	$J_{8,9}$ = $J_{9,10}$ = 11.1
5.48	18	1	m	
5.38 - 5.44	6,8,17	3	m	
5.345	13	1	dd	$J_{12,13}$ = 15.2, $J_{13,14}$ = 7.9
5.29	5	1	m	
3.34	$OC\underline{H}_3$	3	s	
3.05	14	1	dd	$J_{13,14}$ = 7.9, $J_{14,15}$ = 1.9
2.88	7	2	t	$J_{6,7}$ = $J_{7,8}$ = 7.5
2.71	15	1	dt	$J_{14,15}$ = 1.9, $J_{15,16}$ = 5.3
2.21	16a	1	m	
2.16	16b	1	m	
2.07	2	2	t	$J_{2,3}$ 7.3
1.95	4	2	m	
1.91	19	2	m	
1.57	3	2	quintet	$J_{2,3}$ = $J_{3,4}$ = ~7.4
0.87	20	3	t	$J_{19,20}$ = 7.5

Supplemental Table S2: ^{1}H-NMR (600 MHz) in C_6D_6 of 16*S*,17*S*-epoxy-docosahexa-4*Z*,7*Z*,10*Z*,12*E*,14*E*,19*Z*-enoate methyl ester (16,17-Docosatriene A_6 methyl ester)

Chemical shift (ppm)	Proton(s)	Number of protons	Multiplicity	Coupling Constants (Hz)
6.56	12	1	dd	$J_{11,12}$ = 11.0, $J_{12,13}$ = 14.8
6.40	14	1	dd	$J_{13,14}$ = 10.9, $J_{14,15}$ = 15.2
6.10	13	1	dd	$J_{12,13}$ = 14.8, $J_{13,14}$ = 10.9
6.03	11	1	d	$J_{10,11}$ = $J_{11,12}$ = 11.0
5.46	20	1	m	
5.36 - 5.45	5,7,8,10,19	5	m	
5.33	15	1	dd	$J_{14,15}$ = 15.2, $J_{15,16}$ = 7.9
5.29	4	1	m	
3.32	OC$\underline{H}_3$	3	s	
3.06	16	1	dd	$J_{15,16}$ = 7.9, $J_{16,17}$ = 1.9
2.92	9	2	t	$J_{8,9}$ = $J_{9,10}$ = 6.4
2.80	6	2	t	$J_{5,6}$ = $J_{6.7}$ = 6.1
2.71	17	1	dt	$J_{16,17}$ = 1.9, $J_{17,18}$ = 5.3
2.30	3	2	q	$J_{2,3}$ = $J_{3,4}$ = 7.3
2.17	18a	1	m	
2.21	18b	1	m	
2.11	2	2	t	$J_{2,3}$ 7.3
1.91	21	2	quintet	$J_{20,21}$ = $J_{21,22}$ = ~7.4
0.87	22	3	t	$J_{21,22}$ = 7.5
